# Survival and regeneration ability of clonal common milkweed (*Asclepias syriaca* L.) after a single herbicide treatment in natural open sand grasslands

**DOI:** 10.1038/s41598-020-71202-8

**Published:** 2020-08-26

**Authors:** László Bakacsy, István Bagi

**Affiliations:** Department of Plant Biology, Institute of Biology, Faculty of Science and Informatics, University of Szeged, Szeged, 6726 Hungary

**Keywords:** Plant sciences, Ecology, Ecology, Invasive species, Population dynamics

## Abstract

Invasive species are a major threat to biodiversity, human health, and economies worldwide. Clonal growth is a common ability of most invasive plants. The clonal common milkweed *Asclepias syriaca* L. is the most widespread invasive species in Pannonic sand grasslands. Despite of being an invader in disturbed semi-natural vegetation, this plant prefers agricultural fields or plantations. Herbicide treatment could be one of the most cost-effective and efficient methods for controlling the extended stands of milkweed in both agricultural and protected areas. The invasion of milkweed stand was monitored from 2011 to 2017 in a strictly protected UNESCO biosphere reserve in Hungary, and a single herbicide treatment was applied in May 2014. This single treatment was successful only in a short-term but not in a long-term period, as the number of milkweed shoots decreased following herbicide treatment. The herbicide translocation by rhizomatic roots induced the damage of dormant bud banks. The surviving buds developing shoots, growth of the milkweed stand showed a slow regeneration for a longer-term period. We concluded that the successful control of milkweed after herbicide treatment depends on repeated management of treated areas to suppress further spreading during subsequent seasons.

## Introduction

Currently, invasive species are a major threat to biodiversity, human health, and economies^1–4^. It has been estimated that the fight against invasive species and the damage caused by them in European Union accounts for a minimum of 9.6–12.7 billion euros annually, and this amount is expected to rise to 20 billion euros annually^1,5–7^. The most important elements of protection against invasive species are prevention of introduction and early detection. In the case of established invasive species, the most successful options are eradication or isolation^8–13^. Herbicide treatment is one of the most effective ways to control or eradicate invasive plants in large areas^10,14–19^. Nevertheless, herbicide application of invasive species are rather arguable as their application negatively influence the growth of native species, composition of the species and abiotic factors (quality of the above and below ground water supply, soil and air) in protected areas^18,20,21^. Consequently, herbicides have to be carefully chosen (dosage, types and combination) based on the native species community^16,19,20^. Therefore, the herbicide application must be well planned and localized, the applied chemicals should be safe and effective. However, the use of these products in non-agricultural areas are very rarely accessible^10,22,23^. This knowledge gap also requires not only extensive research but also effective exchange of information and experience^10,18,19^.

Clonality is common among invasive plants^24–26^. The common reed *Phragmites australis*^27^, alligator weed *Alternanthera philoxeroides*^28–30^, Japanese knotweed *Fallopia japonica*^31–35^, *Solidago* species^14,36^, and Canada thistle *Cirsium arvense*^37,38^ are examples of problematic invasive clonal species. Their success is partly due to translocation of water, nutrients, and photoassimilates among physically interconnected shoots^39–44^. However, pathogens can also be transported through the same clonal network^38,45–47^ as can heavy metals^48,49^ and herbicides^17,50–53^. Bud banks on a clonal network play an important role in competition, vegetative multiplication, and resprouting^54,55^. An extensive dormant bud bank can be activated, resprouted, and made able to colonize an empty niche or re-establish monospecific stands after disturbance. Subsequently, the succession of natural vegetation can be impeded or completely obstructed. The mortality risk of clonal plants is low because death only occurs when both shoots and bud banks are simultaneously destroyed^40,56–62^. This possibly explains why management of clonal spreading species is difficult even with herbicide treatment^63^, and knowledge of invasive plant biology is essential for effective management^8^. While most studies involved a single year of monitoring, examination of herbicide treatment for several years before, during, and after treatment can provide useful information that will help guide management programs^18,64–67^.

Common milkweed *Asclepias syriaca* is one of the most dangerous invasive transformer species currently widespread in Hungary and is spreading in Czech Republic, Romania, Poland, Serbia, and several other countries^68–72^. It primarily endangers psammophilous habitats where its structure differs from that of natural vegetation^73^. It prefers mostly less heavy soils (well-drained sandy or sandy-loess soils). The colonization of *A. syriaca* can be facilitated by some anthropogenic disturbance of the soils^69^. The problems arising from the invasion of milkweed were primarily attributed to the assumption that it can inhibit the regeneration of natural vegetation^20,69,73,74^. Despite the harmful effects of *A. syriaca*, it was only recently added to the list of Invasive Alien Species of Union Concern^75^. Milkweed originated in North America but is reportedly established in Continental, Mediterranean, and Pannonian Europe^68^. It is a perennial clonal plant^68,69,76,77^, and even though its shoots die back every autumn, it can resprout in the same place for extended periods^69^. The clonal structure of *A. syriaca* comprises solitary or few (2–5) groups of shoots that develop vegetatively by buds of plagiotropic rhizomatic roots^69^. To adequately control common milkweed, the bud banks of its roots and lateral roots must be eliminated. Control or eradication is an increasingly important action from both agricultural and conservation perspectives^20,69,78–80^. Complicating matters is the fact that extermination itself can create suitable conditions for colonization (e.g., soil disturbance), and large areas can become permanently milkweed-free only with coordinated efforts and at enormous costs^69^. Nevertheless, herbicide treatment may be a cost-effective method to control extended stands of milkweed in strictly protected areas^10,20,68,69^. The most frequently used herbicides for *A. syriaca* management are glyphosate and triclopyr, whereas fluroxypyr or dicamba are rarely used. These are often used individually or in combination with each other or with some level of mechanical control^10,20,78,79,81^. Relatively little information is available on the mid-term or long-term effects of post-emergent herbicides on *A. syriaca*. Here we report one of the first and longest monitoring periods of one-time herbicide treatment on a common milkweed stand and analyzed the before, after and during treatment effects. The basic hypothesis of the study is that clonality is an important factor for resistance to herbicide treatment. However, we assumed that a single herbicide treatment influences not only the further spread but also the vegetative and generative propagation of the invasive clonal plant. Therefore, we surveyed the complete shoot network of an isolated common milkweed stand in a long term period. We proposed the following questions: (a) How does the single treatment modify the number of shoots, shoot clusters, and reproductive characters (such as pods and pod-bearing) of common milkweed in the mid-term (three years after application)? (b) How does the stand density change after the single herbicide treatment? Which strategies are used by this invasive species to recover as the stand creates a denser or sparser shoot-network due to re-establish the original area? Based on these results it could be determined how the stand is able to survive the herbicide application. Furthermore, could be determined whether a single herbicide treatment is a successful control measure in the short and mid-term.

## Materials and methods

### Study site

**Figure 1:**
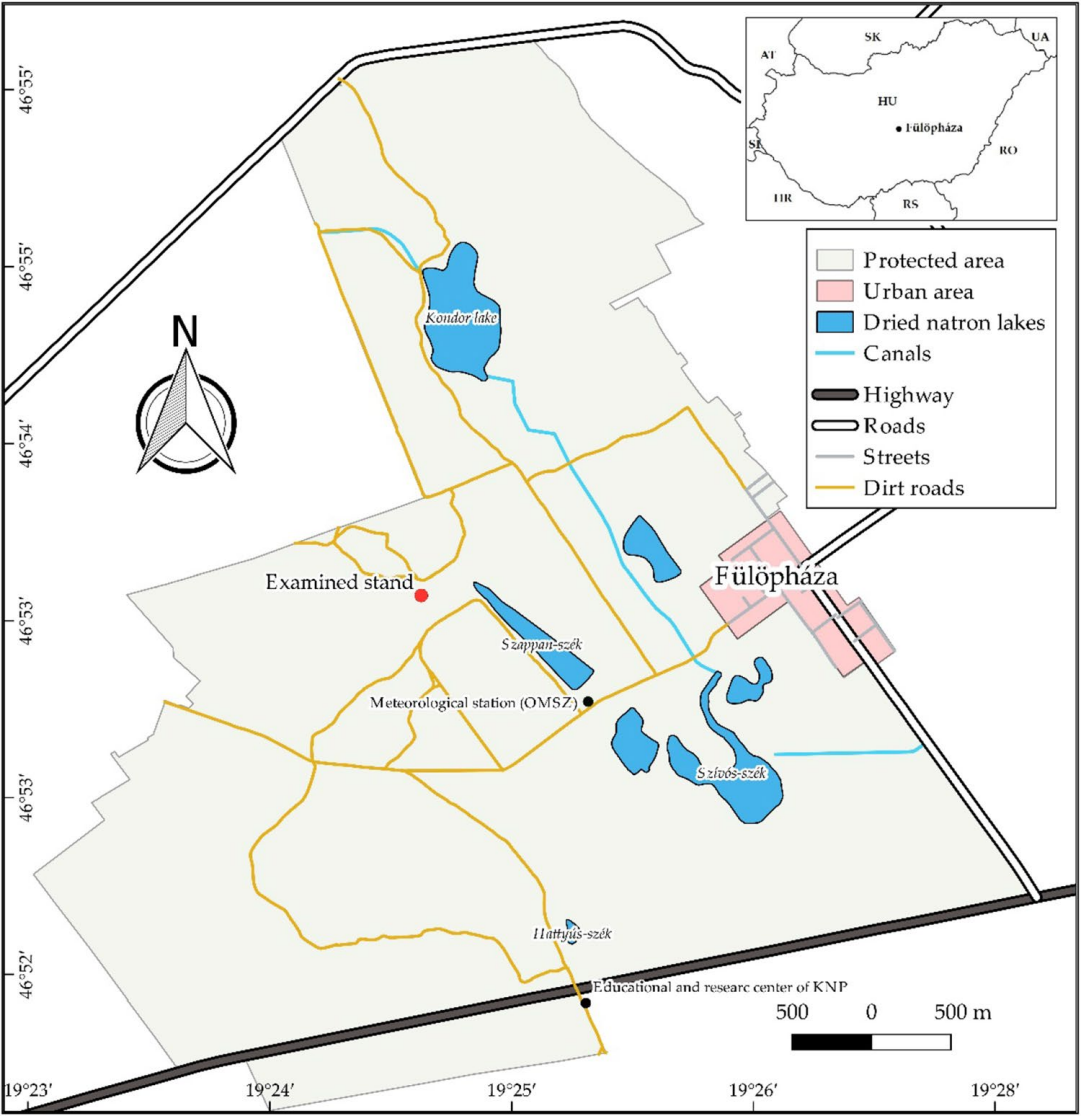
Location of the milkweed stand (red dot). The inserted image in the upper right corner shows the special protected Fülöpháza Sand Dunes, which are a part of Kiskunság National Park in Central Hungary.

The study site is in the UNESCO biosphere reserve, Fülöpháza Sand Dunes in the Kiskunság National Park, Central Hungary (Fig. 1). According to the European Union Habitat Directive (92/43/CEE), Pannonic or open sand steppes (Natura 2000 code: 6,260) represent prominent biomes^82^. Although these dry, nutrient-poor, calcareous sand habitats support only a few communities, many rare, endangered, and endemic species can be found in this area. The site has the following abiotic characteristics: groundwater level is at a high depth^83,84^, mean annual precipitation is 530–565 mm^85–87^, and mean annual temperature is 10.3 °C^87^. As a result, vegetation grows in a mosaic pattern. The 2000-ha study area has been protected from grazing since 1974. In the last quarter-century, the site has been invaded by common milkweed whose extended stands can be found throughout the protected area^20,69,72,73^. In 2011, an isolated milkweed stand embedded in natural psammophilous vegetation units was mapped (GPS coordinates: N46° 53.488′ E019° 24.771′). It had a manageable number of shoots, pods, and stand size (approximately 400 shoots, with a maximum extension of 1,000 m^2^) and was separated from other clones (Fig. 1).

### Herbicide treatment

Herbicide treatment of common milkweed was conducted in the framework of a KEOP tender (KEOP-7.3.1.2-09-2010-0024). The Environment and Energy Operational Program (KEOP) was carried out by the Kiskunság National Park Directorate with the support of the European Union and co-financing from the European Regional Development Fund. This program aimed to suppress the invasive alien plants in the most valuable sand areas of the Danube-Tisza Interfluve. Based on the existing Hungarian practical experiences two methods were applied for the treatment of the target vegetation in the study site: machine broadcast or motor sprayer was applied in buffer areas (formerly arable areas where the target vegetation was very dense), while lubrication (manually) was applied in the more valuable areas (natural vegetations). In the latter case, it can be minimalized the active ingredient to reach the non-target vegetation. The optimal application time was when the target vegetation reached the height of 20–40 cm, until it bloomed (from May to June 2014)^10,88^. Moreover, the study site is rather big and there are some hard-accessible parts. Whereas the examined stand embedded in natural vegetation the lubrication was the applied technology. In this case, the used herbicide was Medallon, within a 50% aqueous solution (2 l ha^−1^). Glyphosate was the active ingredient in Medallon (it is an EPSG synthase inhibitor). Glyphosate belongs to category G of the Herbicide Resistance Action Committee and category 9 of the Weed Science Society of America. The examined stand was treated by herbicide only once (in May 2014) over the 7-year study period (the treatment was not repeated at all). The time of the treatment (phenology) and the used chemical was suitable for a recent study of a basic model for the control of invasive clonal plants^34^.

### Monitoring of herbicide effectiveness for milkweed stand

The investigation extended to the whole stand, and the entire occupied area of the stand was covered with 2 m × 2 m quadrats, in which the localization of the shoots, the number of solitary shoots and clusters (maximum distance between shoots of 15 cm), and pod production of shoots were recorded. The positions of the shoots (to an accuracy of 5 cm) were necessary to depict the pattern of the stand; this allowed the monitoring of individual shoots. The precise location of the shoots was used to calculate a heat map (or Kernel density) to determine the shoot density interpolated over the whole stand. Pod production served as a measure of vitality. The sampling was repeated for 7 years (from 2011 to 2017) in every July. The investigation period was divided as follows: before (the first 3 years), during (hereafter year of treatment, 2014), and after (the last 3 years) treatment.

### Data processing

We used simple data processing methods and basic descriptive statistics to follow the fate of shoots in the stand and demonstrate the efficiency of the herbicide treatment. In this study, deeper statistical analysis (e.g. One-Way ANOVA) was not applicable because it would lead to misleading results due to pseudoreplication^89–94^. GraphPad Prism version 8.0.1.244 for Windows (GraphPad Software, La Jolla, California, USA) was used for calculating descriptive statistics and plotting of diagrams. QGIS version 2.18.24^95^ was used for drawing the study site map, shoot location schemes, and Kernel density analysis.

## Results

A comparison of the before, treatment year and after treatment of the stand clearly shows differences in the heterogeneity and spatial intensity of the shoots.

**Figure 2:**
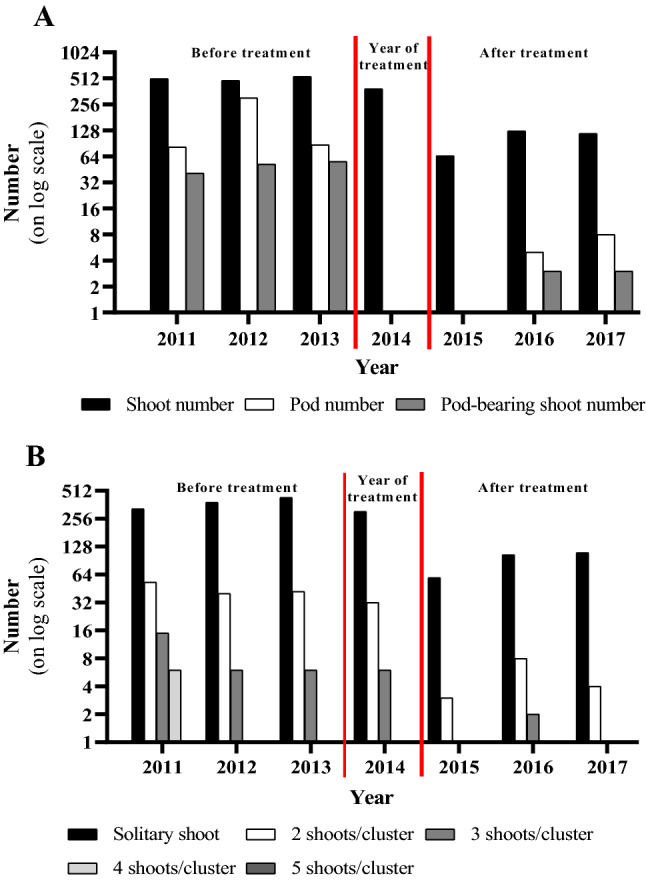
(**A**) Changes in the numbers of shoots, pod-bearing shoots, and pods (**B**) numbers of solitary and clustering milkweed shoots.

**Figure 3:**
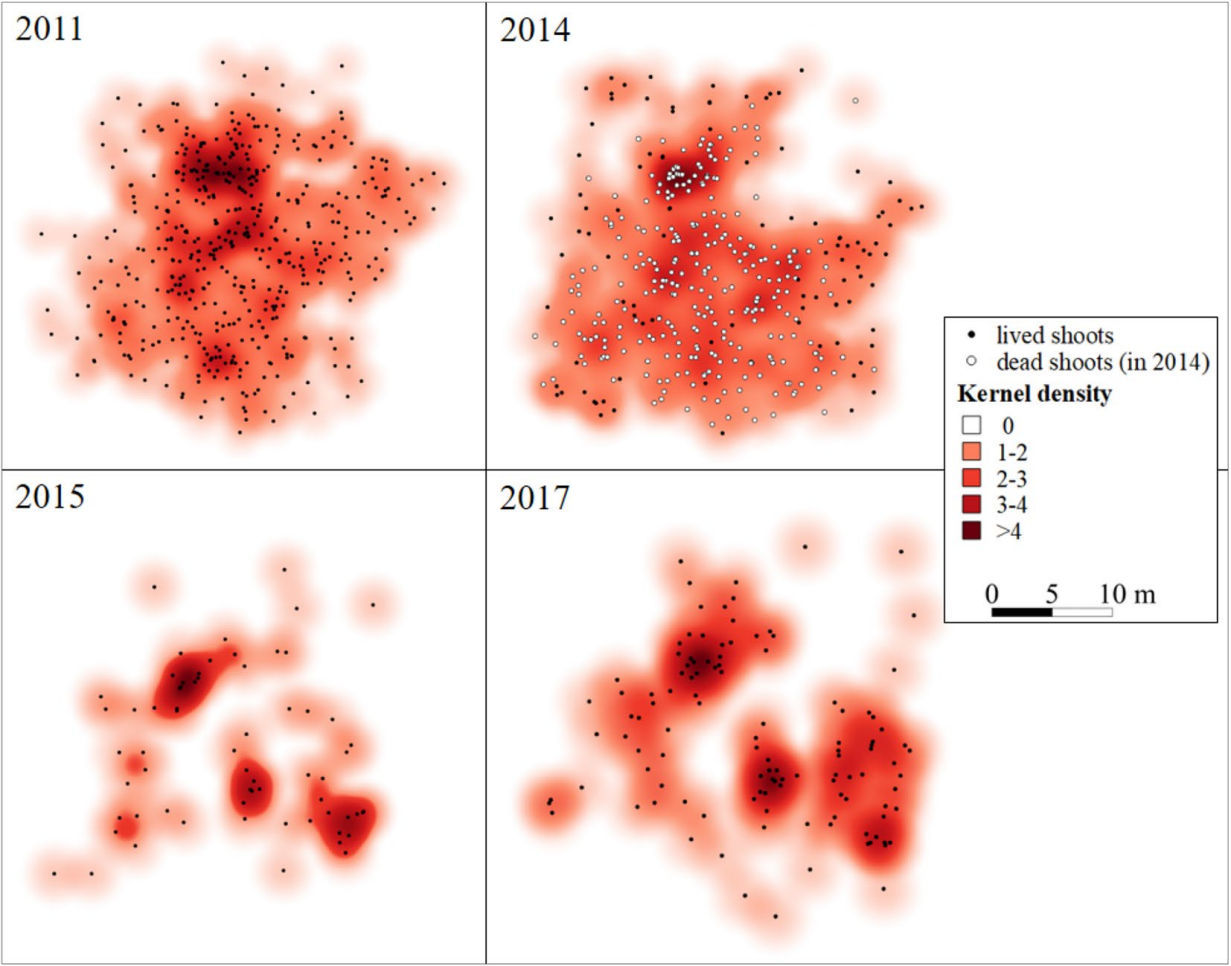
Kernel density of the stand illustrate the arrangement and extension of shoot density changes on the test surface in time: before herbicide treatment (in 2011), the treatment year (2014), and during two years after herbicide treatment (in 2015 and 2017).

Before herbicide treatment, we examined the stand composition from 2011 till 2013 (Figs. 2, 3). This period had the highest shoot number (about 500 shoots) with small fluctuations. In 2012, there was a smaller decrease in the shoot number (22) compared to that in 2011. In the next year (2013), the shoot number increased to 536. The number of solitary shoots increased, whereas that of the more common shoot clusters (2 and 3 shoots/cluster) decreased in the stand. The pod number showed some annual fluctuations in this period with the highest pod numbers (305 pods) in 2012. The annual difference in the number of pod-bearing shoots was small, it increased slightly over the years. The largest increase in the pod-bearing shoot number was in 2012 compared to 2011, but the most pod-bearing shoots (56) emerged in 2013. And it increased by 4 pieces in 2013.

In the year of herbicide treatment (2014), 388 shoots developed, but only 102 of them survived the treatment, but the pod production completely ceased. Due to the effects of the herbicide, 74% of the shoots died, although intact shoots were still observed in almost all areas of the stand (Figs. 2, 3). Solitary shoots were the predominant pattern in the stand (Figs. 2, 3).

After herbicide treatment, we examined the stand composition from 2015 till 2017. While the number of shoots and pods temporarily decreased in the first year after treatment, an increase was observed over the longer-term. A further decrease was observed in the number of shoots in 2015, with almost half of the shoots that have survived from the previous year dying (from 102 survived shoots to 65; Figs. 2, 3). An increase in the number of new shoots and pods was observed from the second year after treatment, although it did not reach its original densities (Fig. 3). The number of solitary shoots increased moderately, whereas the number of those with two or three shoots/cluster decreased after treatment (Fig. 2). The proportion of solitary shoots never decreased below 60%. The solitary shoots represented a higher proportion than in the first period: in 2015, the proportion of the solitary shoots was 90.76%. In 2016, it was 82.53% and in 2017, it was 93.22%. The number of pod-bearing shoots was low: in 2015, there were not pod-bearing shoots, while there were three in 2016 and 2017.

The density of the shoots changed after the treatment; the former dense milkweed stand almost disappeared by glyphosate treatment in 2014, whereas the size of the occupied area by the stand remained almost unchanged, as all the parts remained occupied after treatment (Figs. 2, 3). It is important to note that before and in the year of herbicide application, all the solitary shoots and shoot clusters were recognized forming an extensive and strongly connected, dense stand with one focus point. Compared to the center, the density was lower at the edges of the stand (Fig. 3). After the herbicide treatment, the stand had a lower density of shoots, the shoot clusters vanished, and the stand was fragmented into three smaller isolated foci. The density of these three parts increased after herbicide treatment with sparser edges (Fig. 3).

## Discussion

Our aims were to demonstrate the effects of a single herbicide treatment on the invasive clonal common milkweed. The herbicide treatment influenced the further spread and the trade-off between sexual reproduction and clonal propagation of the invasive common milkweed. There were huge shoot number differences between before and after the herbicide treatment period. Herbicide treatment caused a strong decrease in shoot numbers in the year of the treatment and in the first year after treatment. There are some studies that mechanical control causes a stronger resprouting in *A. syriaca* stands in contrast to our results. One study has reported that one-time cultivation (as a mechanical control) has no effects on milkweed abundance in arable lands^79^. A two years-long study reported that common milkweed shoots resprouted strongly after mechanical control^80^. In the case of the clonal woody *Ailanthus altissima,* Badalamenti et al.^16^ found that more shoots were sprouted after cutting than with combined treatment (herbicide application and cutting). The most likely explanation for this is that dormant bud banks become active in the absence of apical dominance. In accordance with our study, herbicide treatment (even one-time application) is undoubtedly more effective than cultivation or cutting, as reported by Zalai et al.^80^ wherein damage from glyphosate reduced the shoot numbers in the second season (resprouting in their study was half of that in the previous year). Similarly, the resprouted shoots of *C. arvense* were weaker and less dense in the year following herbicide treatment or mechanical control^37^. Moreover, Doğramacı et al.^96^ showed that foliar glyphosate treatment reduced the vegetative growth of *Euphorbia esula* and altered hormone synthesis in crown buds. Saunders and Pezeshki^53^ suggested that changes in leaf and shoot production in *Ludwigia peploides* after herbicide treatment can be a result of translocation-induced hormesis effect. As it was observed in *C. arvense*, glyphosate can translocate across the root toward root buds^97–99^, presumably similar mechanism can occur in the case of *A. syriaca*, because of the similar root system architecture^37,100,101^. When Savini et al.^52^ applied glyphosate to *Fragaria chiloensis* shoots, they found that it was translocated from treated to untreated shoots in the same clone, causing death in both of them. This type of translocation was observed in our study in the year and after the first year of herbicide treatment (2014 and 2015). Therefore, the reduced number of milkweed shoots in this period could be a result of translocated herbicide by rhizomatic roots which could damage dormant bud banks.

The effects of herbicide treatment on reproductive characters (pod and pod-bearing shoots) have been rarely monitored in clonal plants. These reproductive characters play an important role in the spread as well as informs about viability. As a result of herbicide treatment, the pods disappeared in the year of the treatment and the first year after treatment and then they began to recover (Fig. 2). From this data, it can be concluded that sexual reproduction was delayed in the two years after herbicide treatment and only began to increase thereafter, indicating that the stand allocated its resources in favor of clonal growth for survival. This is consistent with numerous observations that sexual reproduction is of secondary importance in clonal plant^102–104^. However, herbicide treatment did not alter the proportions of shoot clusters compared to those before treatment (Fig. 2). It can be concluded that the stand regained its vitality and a one-time herbicide application was not effective to reduce seed production in the longer term. Earlier, Guo et al.^14^ came to a similar conclusion after studying seed production of herbicide treated *Solidago canadensis*. Based on these results, the herbicide treatment could be effective if repeated within the following two years to exterminate invasive clonal plants.

In order to reveal the alterations density and regrowth, Kernel density was used. The Kernel density of the milkweed stand strongly decreased and the stand became fragmented forming three hotspots with shoot clusters due to the single herbicide treatment (Fig. 3). Clonal plants show two main growth forms: phalanx growth form associated with space-occupancy strategy while the guerrilla is a foraging strategy^105^. However, the combined use of these two growth forms has already been shown in many species^35,106,107^. *A. syriaca* has a flexible clonal structure, the plant is able to use denser shoot structure (phalanx type) in favourable habitats and sparser once (guerrilla type) in less suitable habitats^69^. But in this study, the stand did not show any growth in the year of treatment (2014). The guerrilla growth type was typical in 2015 (Fig. 3), showing that this type may serve survival after disturbance. In the next years (in 2016 and 2017), fragments of the stand began to use both growth types again. Our results show that *A. syriaca* used both growth forms (phalanx and guerrilla) for the recovery. The plant is able to use a denser shoot structure for maintaining the formerly occupied area and sparser shoot structure for the colonization of new areas. It seems that the three fragments created new hotspots showing the high spreading potential of the species. It can be concluded that repeated herbicide treatment can be a strategy to prevent milkweed spread.

Based on these results we could determine that one single herbicide treatment of *A. syriaca* stand resulted in severe shoot loss but many shoots could survive indicating that the single treatment was a successful control measure in short but not in the longer-term. Successful survival can be attributed to vegetative propagation, which produces adventitious shoot buds on rhizomatic roots^69,76^ creating a large dormant bud bank. Based on the study of Schmid^108^, the activation of the bud bank in the case of other species depends on clone conditions. This dependence was observed in the case of *A. syriaca*, as several buds appeared on their rhizomatic roots. However, only one or a few of them are activated, whereas the others remain dormant in a given year^109^. The large number of solitary milkweed shoots in the clonal structure shows that the species applies density-dependent regulation to reduce or avoid intraclonal competition. In addition, the shoots will be either vegetative or pod-bearing^110^ as the reproductive output (pod and seed production) is resource limited based on the study of three milkweed species (including *A. syriaca*)^111^. The drastically reduced pod numbers indicate that the pod number as a reproductive output can be a useful indicator for predicting or monitoring the vitality of clonal plants. Successful regrowth of *A. syriaca* benefits greatly from its clonal characteristics and growth after treatment. An extensive dormant bud bank may be activated by disturbance^55,60^. Waldecker and Wyse^112^ found that buds of *A. syriaca* in the proximal part of the rhizomatic root system accumulated less radioactively labeled glyphosate than distal root buds. Therefore, proximal rhizomatic root buds are more dormant than distal ones and thus accumulate less glyphosate. The surviving and newly propagated buds on rhizomatic roots adjusted the numbers of emerging shoots in the years after treatment, and reconstruction of dormant bud banks in 2015 explains the spatial position of the shoots during that period. Our mid-term study demonstrated that a single application of herbicide is successful in the short but not enough on the longer term. The shoot number showed an increase over the longer-term but, the recovery of original shoot numbers in a milkweed stand takes a longer time. The complete destruction of a clone can only be accomplished by methods that affect the entire system of the bud bank and shoots because the mortality risk is distributed among the interconnected bud bank and shoots in a clone^40,56–62^. This indicates that rhizomatous roots and bud banks can create a successful and persistent colonization system, thus making periodic control more effective than a one-time treatment. Thus, in spite of that single herbicide treatment is really suitable for density control in short-term^80^, but it seems the growth of common milkweed stand shows a slow regeneration for a longer-term period.

Furthermore, herbicide treatment raises further questions concerning the native vegetation, especially in nature reserves. We did not observe any visible changes in the vegetation in the year of the treatment and the years after treatment. In contrast to this study, some short-term studies showed that the non-target vegetation was degraded by herbicide application. For example, Gibbson et al.^19^ showed that the phylogenetic diversity of the community became lower due to single herbicide treatment. Szitár and Török^20^ also showed that the single herbicide spraying of common milkweed disturbed the vegetation, and it modified the succession to an earlier initial state. One possible explanation for the absence of non-target side effect is that manual lubrication was applied in the present study, which resulted in a more selective and efficient control method than spraying. These multiple goals require complex and long-term investigations (e.g. pre-invasion conditions are also taken into account).

## Conclusions

In recent years control of invasive species receives a special attention in order to protect natural habitats. The current research surveyed the effect of a single herbicide treatment on one isolated *A. syriaca* stand located in open sand grassland of a protected area. Results of the very fine and temporal replicated sampling method demonstrated that single herbicide treatment modified not just the reproductive output of these invasive species but also their vegetative growth. The clones altered the resource allocation between reproduction and clonal growth in order to survive the herbicide application. The stand became fragmented after the herbicide application generating new hotspots for further clonal spread. Our mid-term case study showed that a large milkweed stand not only can survive a single herbicide treatment but regain its vitality in function of time. Therefore, the first and the second year after herbicide treatment could be optimal period for successful application of further herbicide treatment. The findings of the present study confirm that monitoring of invasive plant control is recommended to be continued for several years after herbicide treatment, especially in the case of clonal plants. Furthermore, long-term examination of the natural vegetation can also broaden our knowledge to clarify how herbicide applications modify natural community. Due to herbicide treatment diminished number of invasive plants might influence the recovery of native species. These results can be useful for plant management of invasive clonal plants or other type of herbicides in the future.

## Data Availability

Data will be available where applicable.
